# Protective Effect of *Cudrania tricuspidata* Extract against High-Fat Diet Induced Nonalcoholic Fatty Liver Disease through Nrf-2/HO-1 Pathway

**DOI:** 10.3390/molecules26092434

**Published:** 2021-04-22

**Authors:** Jitendra Shrestha, Dong-Jae Baek, Yoon-Sin Oh, Sam-Seok Cho, Sung-Hwan Ki, Eun-Young Park

**Affiliations:** 1 College of Pharmacy, Mokpo National University, Jeonnam 58554, Korea; shresthasimon2011@mokpo.ac.kr (J.S.); dbaek@mokpo.ac.kr (D.-J.B.); 2 Department of Food and Nutrition, Eulji University, Seongnam 13135, Korea; ysoh@eulji.ac.kr; 3 College of Pharmacy, Chosun University, Gwangju 61452, Korea; samseok7@gmail.com

**Keywords:** nonalcoholic fatty liver disease, *Cudrania tricuspidata*, antioxidant, nuclear factor, erythroid 2 like 2

## Abstract

Nonalcoholic fatty liver disease is the most common chronic disease affecting a wide range of the world’s population and associated with obesity-induced metabolic syndrome. It is possibly emerging as a leading cause of life-threatening liver diseases for which a drug with a specific therapeutic target has not been developed yet. Previously, there have been reports on the benefits of *Cudrania tricuspidata* (CT) for treating obesity and diabetes via regulation of metabolic processes, such as lipogenesis, lipolysis, and inflammation. In this study, we investigated the ameliorative effect of orally administered 0.25% and 0.5% (*w*/*w*) CT mixed with high-fat diet (HFD) to C57BL/6J mice for 7 weeks. It was found that body weight, fat mass, hepatic mass, serum glucose level, and liver cholesterol levels were significantly reduced after CT treatment. In CT-treated HFD-fed mice, the mRNA expression levels of hepatic lipogenic and inflammatory cytokine-related genes were markedly reduced, whereas the expression level of epididymal lipogenic genes was increased. The mRNA expression level of beta-oxidation and Nrf-2/HO-1 genes significantly increased in CT-treated obese mice livers. We propose that CT alleviates hepatic steatosis by reducing oxidative stress and inflammation.

## 1. Introduction

Nonalcoholic fatty liver disease (NAFLD) is a prime global health concern and affects 25% of the world’s general population [1]. NAFLD, which causes lipid accumulation in the liver, is one of several hepatic disorders caused by hepatic steatosis in patients with no significant history of alcohol consumption, use of steatogenic drugs, or hereditary disorders [2]. Despite considerable previous research efforts, to date, the natural history of NAFLD is not fully understood, although most studies suggest that consuming a high-fat diet (HFD) and a sedentary lifestyle lead to obesity, resulting in the manifestation of metabolic syndromes, hypertriglyceridemia, insulin resistance, and regulation of food intake [3,4,5]. Insulin resistance interferes with lipogenesis and beta-oxidation of fatty acids, disrupting several lipid metabolism-related genes [6]. Pioglitazone is suggested to be beneficial as an insulin sensitizer for treating NAFLD; vitamin E can downregulate some inflammatory-related genes, but effective pharmacotherapeutic remedies for NAFLD remain unknown [7].

Recently, ancient natural products containing phytochemicals, as components with bio-activities such as polyphenols, terpenoids, phenolic constituents and alkaloids, have been investigated for their specific pharmacological effects [8]. *Cudrania tricuspidata* (CT), a thorny tree member of the Moraceae family, is abundant throughout South Asia [9]. The stems and roots of CT have long been used as herbal teas or functional drinks [10]. The fruits, leaves, bark, and roots of CT have been used since ancient times to treat several diseases, such as rheumatism, bruising conditions, scabies, hepatitis, and jaundice. Since the early 21st century, CT extracts, including its flavonoids, xanthones, and organic acids, have been attracting attention for their intriguing biological actions [11]. Various studies have investigated the claims of anticancer [12,13] antimicrobial [14], neuroprotective [15], antiatherosclerotic [16], anti-inflammatory [17], antioxidant [18], antidiabetic [19,20], hepatoprotective [21], and skin-protective [22] abilities of CT and its compounds. Despite these efforts, no study on the effects of CT in NAFLD models has been conducted to date. In this study, we investigated the effects of whole leaf extracts of CT in HFD-fed C57BL/6J mice with respect to alleviative action toward HFD-induced NAFLD.

## 2. Materials and Methods

### 2.1. Cudrania tricuspidata (CT) Extract Preparation

The leaves of CT collected in Sinan-gun, Jeollanam-do, were naturally dried and applied. The dried CT leaves were extracted with hot water at 90–110 °C for 4 h at a ratio of 1:10 (*w*/*v*) to water, cooled to 50 °C, and filtered to remove impurities. The filtered CT extract was concentrated under vacuum pressure at 50–60 °C so that the solid content was 10% to 15% or more. After concentration, dextrin in the same amount relative to the solid content was added (50:50). It was sterilized at 95 °C for 1 h, stirred, and use a spray dryer to dry it under conditions of inlet temperature of 190 °C, an outlet temperature of 95 °C, and a sample supply rate of 12 mL/min.

### 2.2. Chemicals and Reagents

DMEM, fetal bovine serum (FBS), penicillin-streptomycin and trypsin 0.25% were received from GE Healthcare Life Sciences HyClone Laboratories (Pittsburg, PA, USA). Insulin-Transferrin-Selenium solution was received from Gibco Life Technologies Corporation (Grand Island, NY, USA). Kits for detecting blood glucose, triglyceride (TG), and total cholesterol were obtained from Asan Pharm. Co., Ltd (Seoul, Korea). Sodium palmitate, Oleic acid, and kaempferol were received from Sigma Aldrich (St. Louis, MO, USA). Mammalian protein extraction buffer was purchased from GE Healthcare (Chicago, IL, USA). Phosphatase and protease inhibitor cocktail and BCA assay kit were acquired from Thermofisher Scientific (Waltham, MA, USA). Primary antibodies for nuclear factor, erythroid 2 like 2 (Nrf2), β-actin, LaminA/C, HRP anti-mouse, and anti-rabbit secondary antibodies were received from Santa Cruz Biotechnology (Dallas, TX, USA). The primary heme oxygenase-1 (HO-1) antibody was acquired from Enzo Life Science (Farmingdale, NY, USA). cDNA synthesis kit was received from Takara Bio Inc. (Shiga, Kusatsu, Japan). Quantitative real-time PCR (qRT-PCR) primers of sterol regulatory element-binding protein 1c (SREBP-1c), peroxisome proliferator-activated receptor gamma (PPARγ), CCAAT/enhancer-binding protein alpha (C/EBPα), acetyl-CoA carboxylase-1 (ACC1), fatty acid synthase (FAS), tumor necrosis factor alpha (TNFα), hormone sensitive lipase (HSL), carnitine palmitoyltransferase 1a (CPT1a), peroxisome proliferator-activated receptor alpha (PPARα), macrophage marker F4/80, uncoupling protein 1 (UCP-1), HO-1, and nuclear factor, erythroid 2 like 2 (Nrf2) were purchased from Cosmo Genetech (Seoul, Korea). HFD was provided by Research Diets, Inc. (New Brunswick, NJ, USA).

### 2.3. Experimental Animals

A total of C57BL/6J mice (5 weeks, *n* = 34) were purchased from Orient Bio Inc. (Gyeonggi-do, Korea). The study was conducted in accordance with the Declaration of Helsinki, and the protocol was approved by the Ethics Committee of Mokpo National University (MNU—IACUC—2016—012). Mice were acclimatized for one week in a well-maintained animal room with controlled temperature (21–23 °C), humidity (60 ± 2%), 12 h light/dark cycle, free access to food and water.

### 2.4. Induction of Fatty Liver and Treatment with CT Extract

0.25% and 0.5% (*w*/*w*) CT extracts mixed with HFD are manufactured of pellets by Doo Yeol Biotech (Seoul, Korea). After one week acclimatization, mice were randomly divided into four groups as follows. (1) normal chow (NC: 5.4% fat, *n* = 8), (2) HFD (60% fat) group (*n* = 8), (3) 0.25% CT-HFD group (*n* = 9), and (4) 0.5% CT-HFD group (*n* = 9). The fat % was specifically calculated and provided by the manufacturer. Body weights of mice were measured at an interval of a week along with the amount of food consumed. Finally, after 7 weeks, mice were sacrificed and tissues (liver, epididymal fat, subcutaneous fat, and perirenal fat) were collected and weighed.

### 2.5. Cell Culture

Established normal mice hepatocyte cell line AML-12 was received from American Type Cell Collection (ATCC^®^CRL-2254^TM^) (Manassas, MA, USA) and maintained in Dulbecco’s modified eagle’s medium (DMEM) with 10% (*v*/*v*) FBS, penicillin (100 U/mL), streptomycin (100 U/mL) and additionally supplemented with 10 µM insulin, 5.5 µg/mL transferrin, 5 ng/mL selenium and 40 ng/mL dexamethasone. In vitro hepatocellular steatosis of AML-12 cells were induced by exposing cells in high free fatty acid (HFFA) medium supplied with 0.75 mmol/L oleic acid and 0.25 mmol/L palmitic acid conjugated with 1% (*w*/*v*) fatty acid-free bovine serum albumin for 24 h.

### 2.6. MTT Cell Viability Assay

The cells cytotoxicity was assayed using 3-[4,5-dimethylthiazol-2-yl]-2,5 diphenyl tetrazolium bromide (MTT) assay. Briefly, AML-12 cells (4000 cells/well) were grown in 96-well plates and treated with 200 and 400 mg/mL of CT leaf extract, or 50 and 100 µM of kaempferol in the HFFA medium. After 24 h incubation cells were treated with 10 µL EZ-Cytotox (DoGenBio, Seoul, Korea) for 90 min at 37 °C. Absorbance was recorded at 450 nm wavelength in Multiscan GO spectrophotometer (Waltham, MA, USA).

### 2.7. Metabolic Parameters of Blood and Liver Extract

Alanine aminotransferase (ALT), aspartate aminotransferase (AST), TG and total cholesterol were measured using respective kits.

### 2.8. Intraperitoneal Glucose Tolerance Test (IPGTT)

After 6 weeks of CT treatment, mice were deprived of food for 14 h and then a glucose solution (2 g/kg body weight in PBS) was injected intraperitoneally. The blood glucose levels were analyzed using G-Doctor glucometer (Allmedicus, Gyeonggi, Korea).

### 2.9. Histology and Oil-Red O Staining

Liver and epididymal fat tissue sections embedded in paraffin, sectioned at a thickness of 7 μm, and stained with hematoxylin and eosin (H&E) for histological evaluation. For in vitro Oil-Red O staining, AML12 cells were fixed with 10% formalin for 10 min. Cells were stained with Oil-Red O solution for 30 min, followed by washing with 60% isopropanol. Then pictures were captured by LEICA DMi-1 (Wetzlar, HE, Germany) inverted microscope in 20× magnification. After imaging, 100% isopropanol was added into each well and the supernatant was measured absorbance at 500 nm in Multiscan GO.

### 2.10. Estimation of Thiobarbituric Acid Reactive Substances (TBARS) Level for Lipid Peroxidation

Lipid peroxidation in the liver was measured using TBARS [23]. Liver lysate (0.15 mg of liver tissue) was mixed with 300 μL of KH_2_PO_4_ (10 mM) and 1 mL of Tris-HCL buffer (0.15 M, pH 7.4), and incubated with shaking at 37 °C for 20 min. Each 500 μL of 20% TCA and 0.67% thiobarbituric acid solution was added in each sample and boiled at 95 °C in shaking water bath for 15 min than kept into ice-water for 2 min. After centrifugation at 5000 rpm for 10 min, supernatants were placed at 96-well plates and measured the absorbance at 532 nm in Multiscan GO spectrophotometer (Waltham, MA, USA). A standard curve was made by using malondialdehyde as standard at a concentration of 0, 6.125, 12.5, 25, 50, 100 μmol/mL.

### 2.11. qRT-PCR and Analysis of mRNA Expression

RNA of liver tissue, epididymal and brown adipose tissues was extracted using TRIZOL reagent (Invitrogen, Carlsbad, CA, USA). cDNA synthesis was done using PrimeScript 1st strand cDNA synthesis kit according to manufacturer’s protocol (Takara Bio, Shiga, Japan). The different genes of interest were quantitatively analyzed performing qRT-PCR adding SYBR Premix Ex Taq (Takata Bio, Shiga, Japan) and Bio-Rad CFX384 Touch Real-time PCR detection system (Bio-Rad, Hercules, CA, USA) as described by Lin et al. [24]. A total of 40 cycles (2 min at 50 °C, 10 min at 95 °C, and 40 cycles of 10 s at 95 °C and 1 min at 60 °C). Relative copy number was calculated applying threshold crossing point and (C_t_) as calculated by the ΔΔCt calculations.

### 2.12. Western Blot Analysis

The protein expression was determined using western blotting technique [25]. Liver tissue and AML-12 cells were homogenized and mixed in ice-cold mammalian protein extraction buffer added with 1% protease and phosphatase inhibitor cocktail. After 1 h incubation on ice, lysates were centrifuged at 15,000× *g* for 15 min and the supernatant protein samples were collected and quantified using the BCA protein quantification kit using a standard curve. Protein samples were mixed with 5× LDS sample buffer and boiled at 95 °C for 10 min before loading. An equal amount of protein from each sample (20–30 μg per lane) was separated using 8–10% SDS-PAGE, and transferred to PVDF membrane (Immobilon-p, EMD Millipore Corporation, Billerica, MA, USA). After blocking membrane in 3.5% skimmed milk solution at room temperature for 1 h, the primary antibody was treated at 4 °C overnight followed by (HRP)-conjugated secondary antibody for 1.5 h with subsequent washing with 1× TBST buffer. Images were captured in Amersham Imager 680 (GE Healthcare Bio-Science, Pittsburgh, PA, USA).

### 2.13. Fluorescence Microscopy for Analysis of ROS

AML-12 cell (5 × 10^5^/well) were seeded at 12-well plates and incubated for 24 h followed by induction of steatosis and treatment of CT extract or Kaempferol, cells were incubated with 10 µM dihydroethidium (DHE) in serum-deprived medium for 30 min at 37 °C in dark. DHE was discarded, added 1× PBS (1 mL) and photomicrographs were taken with an Olympus fluorescence microscope (Olympus U-LH100HG) at 20× magnification. The image was analyzed and fluorescence intensity was quantified using Image J software.

### 2.14. Statistical Analysis

The in vitro and in vivo data were presented as means ± SD and means ± SE respectively. Non-parametric one-way ANOVA was applied to the data with heterogeneous variance. If the interaction was obtained as significant Turkey post hoc procedure was performed to determine group-wise variation. The difference was considered significant if *p* < 0.05. Graph Pad Prism 7 was used to analyze all the data.

## 3. Results

### 3.1. CT Treatment Reduces the Adiposity and Restores Plasma TG in HFD-Fed Mice

**Figure 1:**
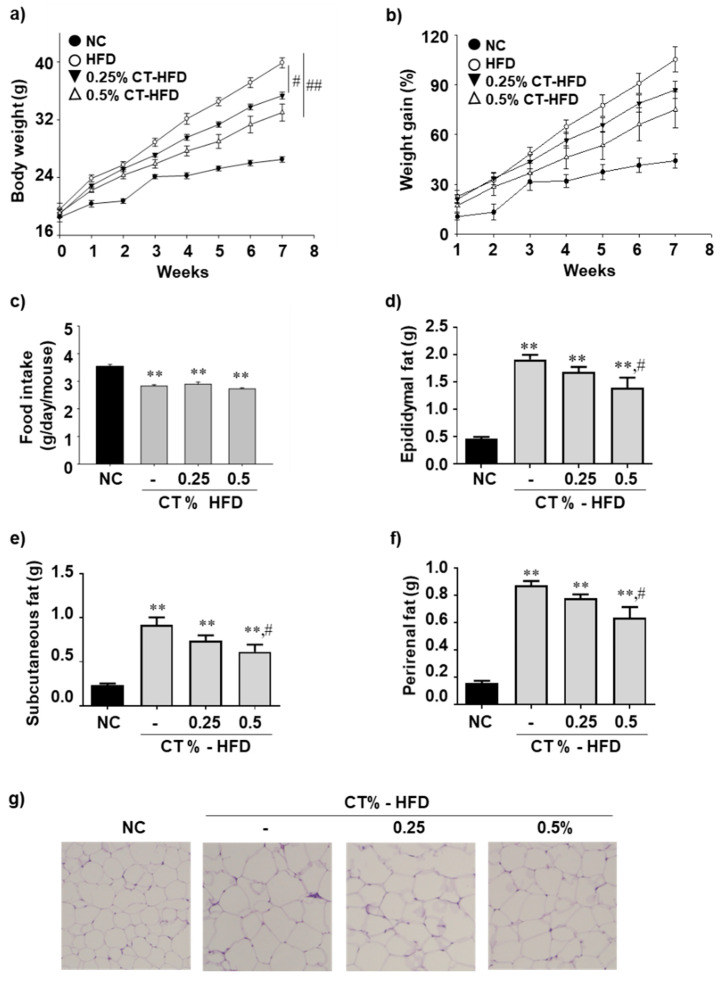
Effect of CT treatment on body weight, fatty tissue mass, and adipose tissue histopathology. One week after acclimatization, C57BL/6J mice were fed HFD mixed with 0.25% and 0.5% CT for 7 weeks. (**a**,**b**) Body weights and weight gain were measured (*n* = 8–9); (**c**) Food intake was monitored weekly; (**d**–**f**) Epididymal, subcutaneous, and perirenal adipose tissues were weighted; (**g**) H&E staining was performed on epididymal adipose tissue sections. NC: normal chow; (-): HFD; CT-HFD; CT treated HFD. Values are mean ± SE. AVOVA *p*-value: ** <0.01 vs. NC group; ^##^ <0.01, ^#^ <0.05, vs. HFD-fed group.

Because NAFLD development is associated with obesity, measuring weight loss is a reasonable approach for assessing its treatment [26]. Therefore, we assessed whether CT treatment affected weight loss in HFD-fed mice. Compared with HFD-fed mice, the body weight significantly decreased by CT treatment (Figure 1a,b). Increasing the CT content from 0.25% to 0.5% was associated with a greater weight loss effect (Figure 1a). There was no difference in the food intake between the HFD-fed group and CT-treated HFD groups, indicating that the CT treatment did not affect the food intake (Figure 1c). Because increased size of adipocytes is a primary indicator of obesity, an important risk factor for NAFLD, comparative evaluation of its size and weight is a useful approach for assessing treatment effectiveness [27,28]. As shown in Figure 1d–f, the epididymal, subcutaneous, and perirenal fat pad weight were significantly lower in HFD-fed mice treated with 0.5% CT than those in HFD-fed mice. Based on H&E staining, epididymal adipocytes decreased in the mice treated with 0.5% CT compared with that in HFD-fed mice. Compared with HFD-fed group, CT treatment improved serum TG as a whole in CT-treated HFD-fed group (Table 1).

### 3.2. CT Treatment Increases the mRNA Expression Level of Epididymal Lipogenic and Beta-Oxidation Genes and Decreases Inflammatory Gene Expression

**Figure 2:**
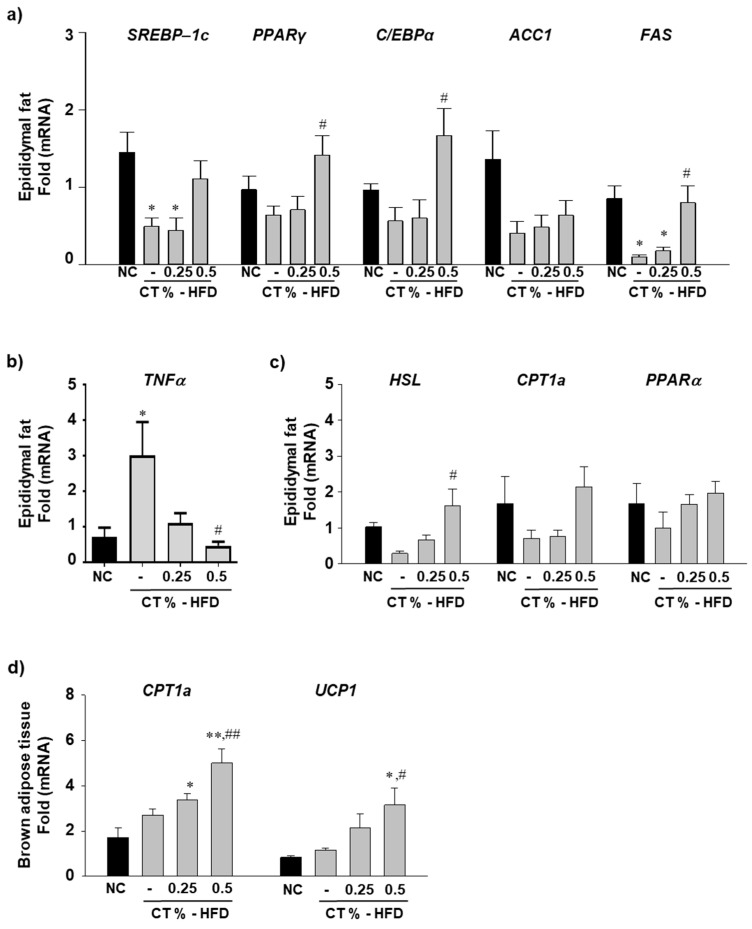
Effect of CT treatment on the metabolic gene expression levels in adipose tissues. One week after acclimatization, C57BL/6J mice were fed HFD mixed with 0.25% and 0.5% CT for 7 weeks. The mRNA expression levels of (**a**) sterol regulatory element-binding protein 1c (SREBP-1c), peroxisome proliferator-activated receptor gamma (PPARγ), CCAAT/enhancer-binding protein alpha (C/EBPα), acetyl-CoA carboxylase-1 (ACC1) and fatty acid synthase (FAS) (**b**) tumor necrosis factor alpha (TNFα) (**c**) hormone sensitive lipase (HSL), carnitine palmitoyltransferase 1a (CPT1a) and peroxisome proliferator-activated receptor alpha (PPARα) were measured in epididymal fat pads by quantitative real-time PCR (qRT-PCR). (**d**) The mRNA levels of CPT1a and uncoupling protein 1 (UCP-1) were analyzed with in brown adipose tissues by qRT-PCR. NC: normal chow; (-): HFD; CT-HFD; CT treated HFD. Values are mean ± SE. AVOVA *p*-value: ** <0.01, * <0.05 vs. NC group; ^##^ <0.01,^#^ <0.05 vs. HFD-fed group.

To measure the mRNA expression level of adipogenesis-related genes, we performed qRT-PCR analysis of mRNA obtained from epididymal fat. Major lipogenesis-related genes, such as SREBP-1c, PPARγ, C/EBPα, ACC1, and FAS mRNA levels were reduced in the HFD-fed group than those of compared with NC group (Figure 2a). However, treatment with 0.5% CT increased the mRNA expression level of SREBP-1c, PPARγ, C/EBPα, and FAS (Figure 2a). Because increased expression of inflammation-related genes of epididymal fat is a major initial biomarker of obesity-induced NAFLD in HFD-fed mice [29], we assessed whether CT treatment decreased inflammatory cytokine-related gene expression in HFD-fed group. Inflammatory gene *TNFα* levels were significantly higher in the HFD group than those in the NC group and notably downregulated in the HFD groups treated with 0.5% CT compared with HFD groups (Figure 2b). Fatty acid oxidation is a vital pathway for energy burning in mice [30]. The levels of fatty acid oxidation-related gene expressions such as HSL, CPT1a and PPARα were lower in the epididymal fat of the HFD-fed group than those in the NC group. Treatment with 0.5% CT increased mRNA expression levels of HSL (*p* < 0.05), CPT1a, and PPARα compared with HFD-fed groups (Figure 2c). Brown adipocytes are considered beneficial in animal as they uncouple ATP by mitochondrial respiration, dissipating a sufficient amount of heat in the body [31]. We found that treatment with 0.5% CT significantly increased the CPT1a and UCP1 mRNA expression levels in the CT-treated groups compared with NC and HFD-fed groups (Figure 2d).

### 3.3. Blood Glucose Level and Glucose Tolerance Improved by CT Supplementation

**Figure 3:**
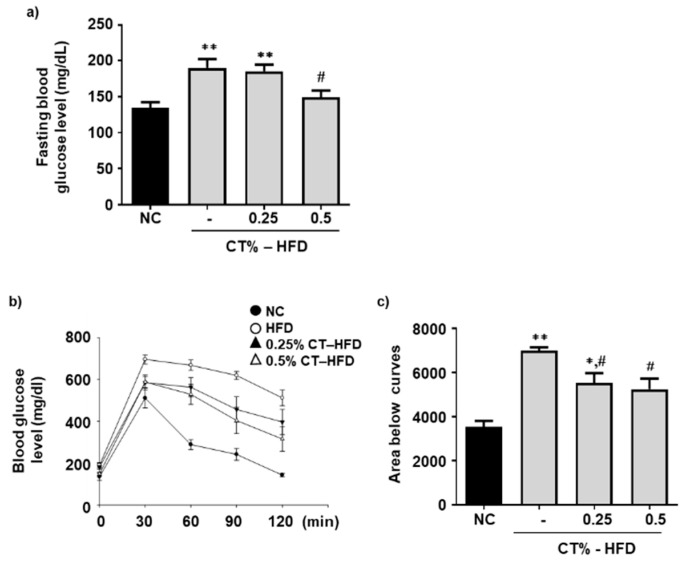
Effect of CT treatment on serum glucose level and glucose tolerance. One week after acclimatization, C57BL/6J mice were fed HFD mixed with 0.25% and 0.5% CT for 7 weeks. (**a**) At 6 weeks of treatment, blood glucose levels were measured in mice fasted overnight (NC group: *n* = 4; HFD only, 0.25% and 0.5% treated with HFD group: *n* = 8). (**b**) Glucose tolerance test of all group was performed. (**c**) Area below curve were plotted. NC: normal chow; (-): HFD; CT-HFD; CT treated HFD. Values are mean ± SE. AVOVA *p*-value: ** <0.01, * <0.05 vs. NC group; ^#^ <0.05 vs. HFD-fed group.

Elevated blood glucose levels and insulin resistance are the initial indicators of obesity-induced type 2 diabetes mellitus, a promising risk factor of NAFLD [32]. The fasting blood glucose levels of all mice groups were measured. Fasting blood glucose levels were significantly higher in the HFD-fed group than those in the NC group and decreased in the HFD-fed treated with 0.5% CT compared with the HFD group (Figure 3a). Comparative IPGTT were conducted in all four groups to investigate the effect of CT on glucose tolerance improvement after glucose administration. Blood glucose levels were significantly lowered in both HFD groups treated with 0.25% and 0.5% CT (Figure 3b). The area under the curve plotted for the IPGTT revealed that CT treatment notably improved glucose tolerance (Figure 3c).

### 3.4. Effect of CT Extract on Hepatic Fat Accumulation, Lipogenesis, Beta-Oxidation and Inflammation in HFD Fed Mice

**Figure 4:**
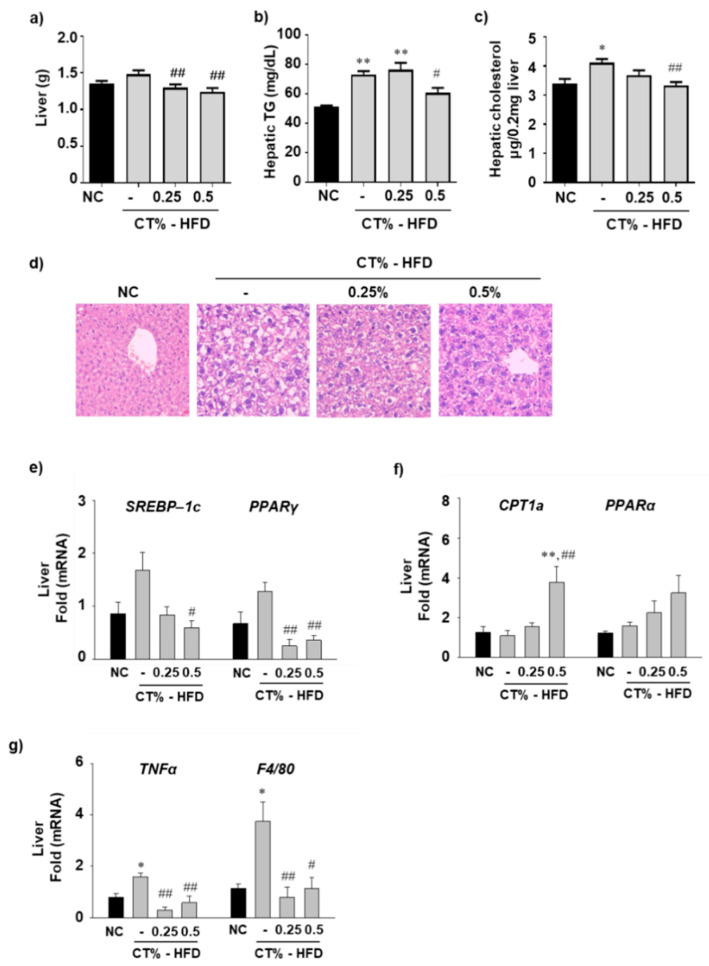
Effect of CT treatment on hepatic steatosis. One week after acclimatization, C57BL/6J mice were fed HFD mixed with 0.25% and 0.5% CT for 7 weeks. After 7 weeks, (**a**–**c**) Liver mass, hepatic TG level and hepatic cholesterol levels were measured. (**d**) H&E staining was performed on liver sections. The mRNA levels of (**e**) sterol regulatory element-binding protein 1c (SREBP-1c) and peroxisome proliferator-activated receptor gamma (PPARγ) (**f**) carnitine palmitoyltransferase 1a (CPT1a) and peroxisome proliferator-activated receptor alpha (PPARα) (**g**) tumor necrosis factor alpha (TNFα) and macrophage marker F4/80 were analyzed in liver by RT-qPCR. NC: normal chow; (-): HFD; CT-HFD; CT treated HFD. Values are mean ± SE. AVOVA *p*-value: ** <0.01, * <0.05 vs. NC group; ^##^ <0.01, ^#^ <0.05 vs. HFD-fed group.

Hepatic fat accumulation and hepatocellular hypertrophy are the hallmarks of NAFLD [33]. To investigate the effect of CT treatment on liver hypertrophy, the liver weights in all mice groups were compared. Liver weight was higher in the HFD-fed group than that in the NC group, and CT administration at both concentrations was associated with significantly reduced liver weights (Figure 4a). Consequently, hepatic TG and cholesterol levels were significantly higher in the HFD group than those in the NC group; however, the hepatic TG and cholesterol levels were significantly lower in the HFD-fed group treated with 0.5% CT than those in the HFD-fed group (Figure 4b,c). H&E staining of liver sections revealed that lipid droplets in the HFD-fed groups treated with 0.25% and 0.5% CT were significantly lower than those in the HFD-fed group (Figure 4d). In our study, reduced TG and cholesterol levels and hepatic fat accumulation in CT-treated HFD mice could be regulated via increased beta-oxidation of free fatty acids (FFAs), reduced influx of non-esterified FFAs, and decreased synthesis of lipids [34]. Therefore, we investigated whether CT administration regulates hepatic lipogenic gene expression in liver tissue. The mRNA expression levels of SREBP-1c and PPARγ were significantly lower in the HFD-fed group treated with 0.5% CT than those in the HFD-fed mice (Figure 4e). To determine the expression levels of genes related to fatty acid oxidation, we examined the mRNA expression level of CPT1a and PPARα. The mRNA expression levels of CPT1a were significantly increased in the HFD-fed group treated with 0.5% CT compared with the NC and HFD groups, and PPARα tended to increase in the CT-treated HFD-fed group but not to a significant extent (Figure 4f). Because inflammation is a major risk factor for NAFLD in mice [35], we also analyzed hepatic inflammatory gene expression levels. The mRNA expression levels of TNFα and F4/80 were significantly increased in the HFD group compared with the NC group and decreased in the HFD group treated with 0.25% and 0.5% CT compared with the HFD group (Figure 4g).

### 3.5. Antioxidant Role of CT Extract in Protecting Hepatocytes from HFD-Induced Hypertrophy through Nrf2/HO-1 Mediated Pathway

**Figure 5:**
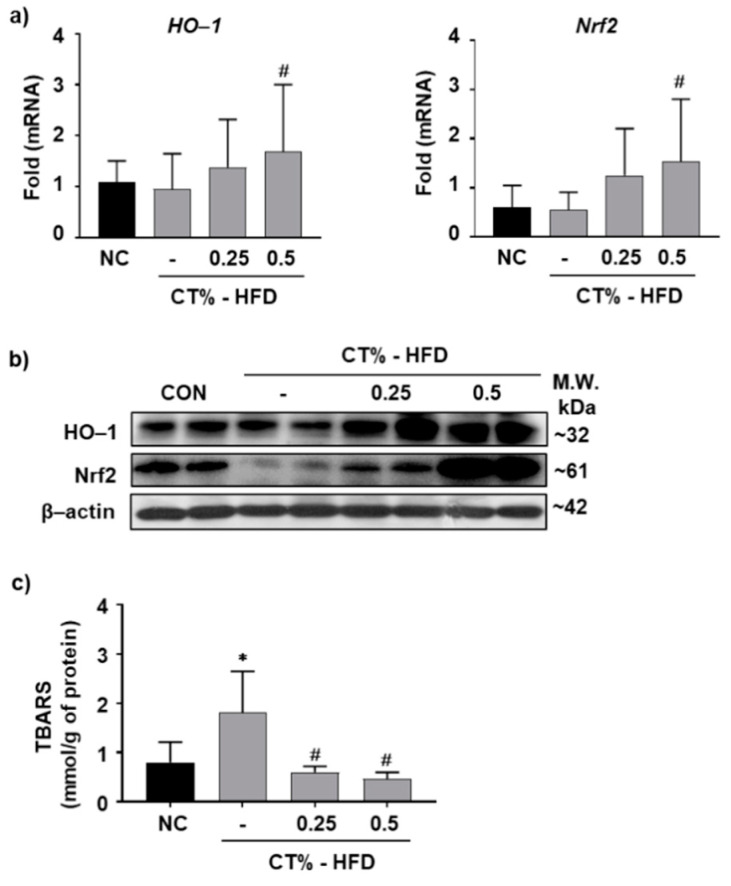
Antioxidant effect of CT on HFD-fed mice. One week after acclimatization, C57BL/6J mice were fed HFD mixed with 0.25% and 0.5% CT for 7 weeks. The liver mRNA expression level of (**a**) heme oxygenase-1 (HO-1) and nuclear factor, erythroid 2 like 2 (Nrf2) were analyzed by RT-qPCR. (**b**) The protein expression levels of HO-1 and Nrf2 were estimated by western blotting and normalized to β-actin. (**c**) The level of thiobarbituric acid reactive substances (TBARS) were analyzed in liver lysate. NC: normal chow; (-): HFD; CT-HFD; CT treated HFD. Values are mean ± SE. AVOVA *p*-value: * <0.05 vs. NC group; ^#^ <0.05 vs. HFD-fed group.

Oxidative stress is considered as the etiology of NAFLD by its “second hit” action, and the Nrf2/HO-1 pathway is a major defense mechanism against oxidative stress [36]. We examined the expression levels of HO-1 and Nrf2, the key transcription factors for HO-1 regulation in all groups. The mRNA expression levels of HO-1 and Nrf2 were significantly increased in the HFD-fed mice treated with 0.5% CT than those in the HFD group (Figure 5a). The protein expression levels of HO-1 and Nrf2 was induced according to the dose of CT administered in the HFD group compared with the HFD-fed group (Figure 5b). To analyze whether CT treatment had an antioxidant effect, TBARS, the end product of lipid peroxidation in liver tissues, were quantified. The TBARS level was significantly higher in the HFD group than that in the NC group and lower in CT-treated HFD-fed group than that in the HFD group (Figure 5c). These results showed that CT extracts have a prominent antioxidant effect in HFD-fed mice and may alleviate HFD induced liver injury.

### 3.6. CT Extract Attenuates In Vitro Lipid Accumulation in Mice Hepatocyte AML-12 Cells

**Figure 6:**
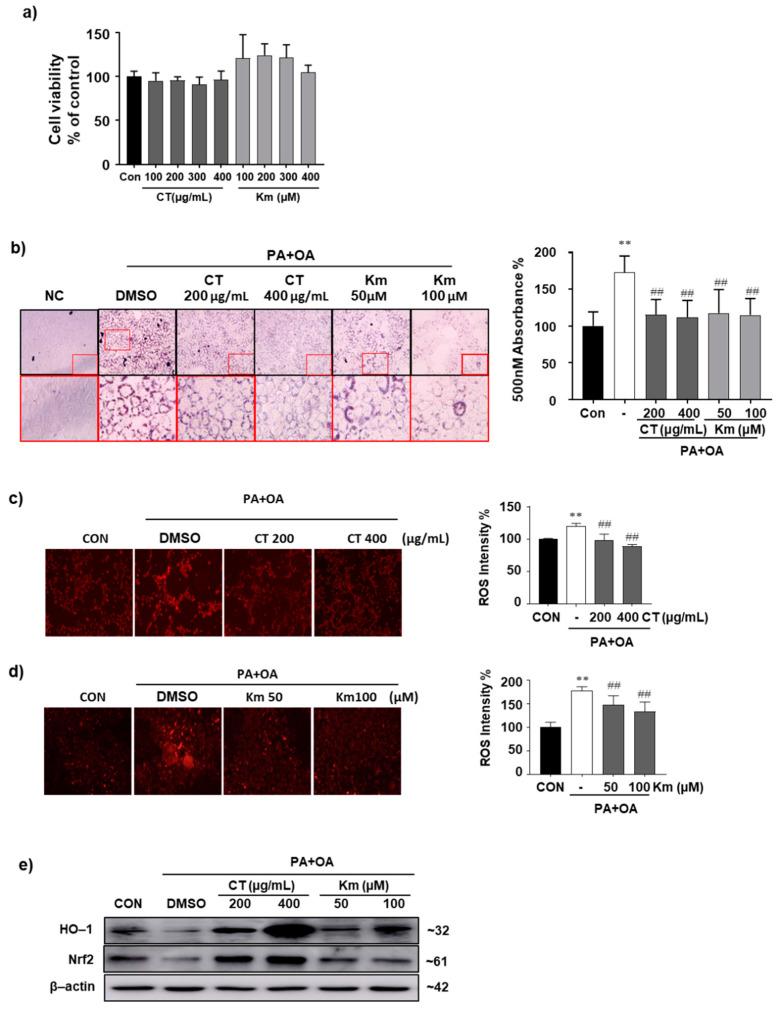
CT extract reduces in vitro lipid accumulation in AML-12 mice hepatocyte cells. (**a**) MTT assay was performed in AML-12 cell treated with whole CT leaf extract (100–400 µg/mL) and kaempferol (100–400 µM). (**b**) AML-12 cells were seeded, after inducing steatosis by 1 mmol/L (3:1, oleic acid: palmitic acid) medium, cells were treated with 0.1% DMSO or CT extract (200 and 400 µg/mL) or kaempferol (50 and 100 µM) and performed Oil Red O staining (left) and absorbance (right) was observed at 500 nm. Intracellular ROS was estimated by dihydroethidium (DHE) staining in AML12-cell treated with (**c**) CT extract (**d**) kaempferol and bar graph was plotted for fluorescence intensities of DHE (each right side). (**e**) The expression level of heme oxigenase-1 (HO-1) and nuclear factor, erythroid 2 like 2 (Nrf2) were performed by western blotting and normalized to β-actin. The result is representative of three individual experiments. Values are mean ± SD. AVOVA *p*-value: ** <0.01 vs. control group; ^##^ <0.01 vs. DMSO treated group.

Kaempferol is a major active flavonoid found in a variety of plants, including CT [11]. It has various pharmacological effects, including antioxidant effects in various cell lines [37,38]. To determine whether CT extract and its major flavonoid kaempferol are involved in improvement of lipid accumulation in liver, an in vitro experiment was conducted using AML12 cells. Initially, we examined the cytotoxic effects of CT extract and kaempferol on AML-12 cells. Working concentrations of both CT extract and kaempferol were noncytotoxic to AML-12 cells (Figure 6a). To determine whether CT extract and kaempferol treatment in hepatocytes affected the inhibition of fat accumulation, AML-12 cells were stained with Oil Red O after intracellular fat deposition with palmitic acid (PA) and oleic acid (OA). Lipid accumulation in groups treated with PA and OA increased by 1.72-fold compared with the control group, whereas treatment with 200 and 400 µg/mL CT significantly reduced lipid accumulation by 0.66- and 0.64-fold, respectively, compared with the vehicle-treated group (Figure 6b). Treatment with kaempferol (50 and 100 µM) also reduced lipid accumulation in hepatocytes by 0.68- and 0.66-fold, respectively, compared with the vehicle-treated group (Figure 6b).

### 3.7. CT Extract Attenuates Reactive Oxygen Species in AML-12 Cells by Inducing Nrf2 and HO-1 Expression

In animal study, we observed the alleviating effect of CT extract in improving HFD induced NAFLD through increasing expression of antioxidant related proteins. To evaluate the effects of CT extract and kaempferol on reactive oxygen species (ROS) production, we performed DHE staining in AML-12 cells treated with 200 µg/mL and 400 µg/mL of CT extract or 50 µM and 100 µM of kaempferol after inducing steatosis by OA and PA (Figure 6c,d). In vitro ROS production was significantly higher in the OA and PA treatment groups than that in the control group. ROS production was significantly reduced on treatment with CT extract (Figure 6c) and kaempferol (Figure 6d) in a dose-dependent manner. To determine how ROS production was reduced in response to treatment with CT extract and kaempferol, we performed immunoblotting of cell protein lysate for antioxidant-related genes. As shown in Figure 6e, treatment with OA and PA in AML-12 cells significantly reduced the expression level of Nrf2; consequently, the activity of downstream enzyme, HO-1, was also reduced markedly. Nrf2 expression was elevated by CT and kaempferol treatment in AML-12 cells. The expression level of HO-1, a protein downstream of Nrf2, was increased in cells treated with 200 µg/mL and 400 µg/mL of CT extract and in kaempferol-treated cells compared with vehicle-treated cells. These results shows that, CT extracts shows antioxidant properties by neutralizing free fatty acid induced ROS production by increasing expression of antioxidant related genes. Furthermore, kaempferol might be the possible major flavonoid of CT extract responsible for showing antioxidant properties.

## 4. Discussion

Despite considerable research efforts, the pathophysiology of NAFLD remains unclear, making it more difficult to identify specific therapeutic targets for this disease. Previous suggests that consuming an HFD and a sedentary lifestyle contribute to obesity and lead to the manifestation of metabolic syndrome, hyperlipidemia, and insulin resistance through the deteriorative performance of several inflammatory cytokines and interruptions in natural metabolic pathways [3,4]. Hyperlipidemia causes adipocyte hypertrophy and fat accumulation in extra-adipose tissues such as liver and muscle tissues, leading to increased oxidative stress and release of cytokines, such as TNFα, interleukin (IL)-6, and IL-1β [31]. Abnormalities in liver lipid metabolism and storage sites owing to obesity result in NAFLD, a condition of hepatic fat deposition accompanied by inflammation and cellular injury. Suppressing progressive NAFLD is described as an effective approach for treating chronic liver diseases. Numerous drugs have been studied to treat NAFLD, but clinically available drugs are limited. Therefore, natural products have been intensively investigated as possible alternatives for preventing and treating NAFLD. In this study, we found that CT treatment improved excess hepatic fat accumulation, liver hypertrophy, and lipid profiles in an HFD-induced liver injury model.

Body weight control plays a significant role in controlling NAFLD by reducing the risk of developing obesity. Reduction in the body weight by CT treatment may be owing to metabolic control rather than loss of appetite. Reduced body weight is attributable to the inhibition of pancreatic lipase, independent of appetite alteration [19,20]. Ethanol extract of CT has been reported for the inhibition of pancreatic lipase [39]. CT treatment seems to decrease the availability of free fatty acid in small intestine, therefore improve serum lipid profile, which is the major cause of fatty liver. In this study, it was also observed that CT treatment improved the plasma TG level compared to HFD-fed treated group. In addition, inflammation due to increased white fat mass (WAT) such as epididymal, perirenal, and subcutaneous fat exacerbates hepatosteatosis in HFD-fed mice. Treatment with CT extract decreases the expression levels of inflammatory genes and increases the expression levels of genes related to fatty acid oxidation, thereby reducing the mass of WAT and demonstrating a positive role in improving the deterioration of the fatty liver.

According to the hit hypothesis of NAFLD pathogenesis, oxidative stress is involved in the second-hit action, wherein a fatty liver worsens with nonalcoholic steatohepatitis (NASH) and hepatic fibrosis [40]. The elevated levels of ROS, owing to mitochondrial dysfunction, lead to inflammation, hepatic stellate cell activation, and lipid peroxidation, resulting in the deposition of fibrotic collagen in the liver. Lipid peroxidation and pro-inflammatory proteins are indicators of NASH progression in HFD-fed mice [40]. In the present study, an elevated TBARS level in HFD-fed mice is indicative of increased oxidative stress in liver tissues. The by-products of lipid peroxidation are intensely cytotoxic and are diffusible into extracellular spaces which can cause extrahepatic cell death [41]. In this study, administration of CT extract improved the antioxidant status by enhancing antioxidant enzyme activities and restoring the lipid peroxidation level, which protects hepatocytes from HFD-induced oxidative stress. Nrf2 is a key regulator of antioxidant response to oxidative stress, coupled with the Keap1 enzyme. Nrf2 released by the degradation of Keap1, is translocated into the nucleus to form transactivation complexes on an antioxidant response element (ARE), which promotes the transcription of various antioxidant enzymes. HO-1 is a major ARE-inducible antioxidant enzyme that catalyzes heme degradation to produce carbon monoxide (CO), ferrous ion, and biliverdin. CO modulates inflammatory reactions by specifically regulating inflammatory cytokine level, whereas biliverdin acts as an antioxidant to prevent lipid peroxidation [42]. In our study, administration of CT extracts increased the expression level of total Nrf2 and induced the expression level of its downstream target HO-1 in a dose-dependent manner. The result of lipid peroxidation end-product quantification (TBARS) showed that CT extract exerted an antioxidative effect on the HFD-fed mice. Kaempferol-7-O-β-D-glucopyranoside is a major flavonoid compound found in leaf extracts of CT and is a prominent ROS scavenger [43]. To further validate the effects of CT extract and kaempferol, we performed an in vitro experiment in normal hepatocyte AML-12 cells by treating them with PA and OA, which are models for increasing hepatocyte fat accumulation [44]. CT extract and kaempferol reduced the lipid droplets in AML-12 cells, which is similar to the findings of previous reports showing an ameliorative effect of CT extract in lipid droplet accumulation in 3T3 adipocytes [19]. CT extract and kaempferol significantly increased the expression of total Nrf2 and HO-1, leading to reduced generation of intracellular ROS (as assessed by DHE staining). The stronger ROS-scavenging properties and higher antioxidant gene expression levels in CT-treated cells than in kaempferol-treated cells suggest that other antioxidant components are also present in CT leaf extracts besides kaempferol. CT leaf extracts exhibited higher antioxidant properties than extracts of other parts of the plant and contained flavonoid quercetin along with kaempferol [11]. Previous studies have shown that isoflavonoids and cudraflavone-A, isolated from the roots of CT, increase nuclear translocation of Nrf2 and expression of the HO-1 gene in BV2 mice cells [45]. In addition, CT leaf extracts reportedly protect hepatocytes from oxidative stress by suppressing the cytochrome P450 2E1 enzyme [46]. Although various active ingredients contained in CT inhibit oxidative stress in hepatocytes by various mechanisms, further exploration is needed on how major CT extract–related active components degrade the Nrf2-Keap1 complex and promote nuclear translocation of Nrf2.

## 5. Conclusions

The results of the present study revealed that long-term consumption of an HFD produces sequential obesity and glucose tolerance, resulting in the histological and metabolic condition of nonalcoholic fatty liver. Oral administration of CT could protect against NAFLD development in HFD-fed mice in a dose-related manner, as evidenced by decreased body weight and glucose levels, improved liver/BAT histology, and restored metabolic markers of NAFLD. The ameliorative effect of the supplements of CT extract can be attributed to the marked diminution of liver weight/WAT, improved lipid metabolism, and decreased number of hepatic/adipocyte pro-inflammatory cytokines. In the present study, the reduction of oxidative stress in the liver through increased Nrf2/HO-1 expression of CT extract suggesting that CT extract can be used for preventing and managing of western diet-related chronic diseases, including type 2 diabetes mellitus and NAFLD. CT extract can be developed as functional foods and its active components can be used to make pharmaceutical components for the management of oxidative stress-related metabolic disease. Further investigations are needed to clarify in vivo effect of active components in NAFLD animal models.

## Figures and Tables

**Table 1:** Change of blood chemistry in C57BL/6J mice for 7 weeks of HFD-fed group and HFD-fed treated with CT.

Treatment	Triglyceride	Cholesterol	AST	ALT
NC	138.2 ± 11	135.8 ± 10	133.0 ± 6.4	38.8 ± 2.50
HFD	189.3 ± 40 **	170.3 ± 12 **	146.5 ± 8.3	40.3 ± 3.41
0.25% CT-HFD	151.9 ± 13 ^#^	179.5 ± 8 **	111.3 ± 15.2 ^#^	32.9 ± 2.01
0.5% CT- HFD	151.2 ± 21 ^#^	183.1 ± 10 **	124.6 ± 10.6	31.4 ± 0.62

NC: normal chow; (-): HFD; CT-HFD; CT treated HFD. Values are mean ± SE. AVOVA *p*-value: ** <0.01 vs. NC group; ^#^ <0.05 vs. HFD group.

## Data Availability

Not applicable.

## Sample Availability

Samples of the compounds are not available from the authors.
